# A Wide Spectral Range Reflectance and Luminescence Imaging System

**DOI:** 10.3390/s131114500

**Published:** 2013-10-25

**Authors:** Tapani Hirvonen, Niko Penttinen, Markku Hauta–Kasari, Mika Sorjonen, Kai–Erik Peiponen

**Affiliations:** 1 Institute of Photonics, University of Eastern Finland, P.O.Box 111, FI–80101 Joensuu, Finland; E-Mails: npenttin@uef.fi (N.P.); markku.hauta-kasari@uef.fi (M.H.-K.); kai.peiponen@uef.fi (K.-E.P.); 2 Palomäentie 4, FI–88620 Korholanmäki, Finland; E-Mail: sorjonen.mika@gmail.com

**Keywords:** hyperspectral sensors, hyperspectral imaging, luminescence, 78.40.-q, 78.60.Lc, 42.79.Pw

## Abstract

In this study, we introduce a wide spectral range (200–2500 nm) imaging system with a 250 μm minimum spatial resolution, which can be freely modified for a wide range of resolutions and measurement geometries. The system has been tested for reflectance and luminescence measurements, but can also be customized for transmittance measurements. This study includes the performance results of the developed system, as well as examples of spectral images. Discussion of the system relates it to existing systems and methods. The wide range spectral imaging system that has been developed is however highly customizable and has great potential in many practical applications.

## 1. Introduction

Spectral information has been widely used in research for several decades. This popularity is based on the contactless method by which to sense the properties and state of the observed system. The usage of the wide wavelength (*λ*) range of the electromagnetic spectrum makes it possible to observe different properties of the target system. These include, for example, identifying the mechanical properties of wood by using near-infrared (NIR) radiation [1] and classifying wood types based on a fluorescence spectrum [2]. There are many other potential applications for the use of spectral methods, for example, in the food industry and in biological research.

There are commercial devices available for spectral data acquisition of the 175–3300 nm wavelength range, but those systems are usually only capable of measuring from one point at a time. For example, there exists even simple spectrophotometers in that range. However, imaging systems have a great advantage compared to single point measurement devices, because they can measure thousands of points at the same time. These points produce a so-called spectral image, where each spatial pixel has its own spectrum. From the spectral image, it is possible to observe the spatial distributions of heterogeneous samples and, thus, calculate a two-dimensional (2D) map of some specific feature. Conventionally, these imaging systems do not have such a wide spectral range when compared to the point measurement systems, as shown in Table 1, and this limits practical applications. According to available resources, the widest spectral range of 405–3000 nm in imaging has been reported by Pieters *et al.* (2009), with their remote sensing system of the Moon [3]. Spectral Imaging Ltd. (2012) has achieved 2500 nm at the infrared end of the spectral range, and Heia *et al.* (2007) have achieved 350 nm as the shortest wavelength [4,5]. Spectral resolutions of all systems vary between 2–16 nm, whereas spatial pixels (px) in sensors are limited between 290–2048 pixels. Spatial resolutions depend on the geometry of the imaging setup, and values are from 60 μm to 70 m.

Existing wide spectral range imaging systems (presented in Table 1) are not able to operate properly in the ultraviolet (UV) region of the electromagnetic spectrum. Furthermore, these systems are not reported to support luminescence imaging. These limitations may be critical in practical applications, and thus, a system improvement is needed.

In this study, we introduce a UV and luminescence improved 200–2500 nm spectral imaging system. The setup is based on three line spectral cameras and can be freely modified. The system testing has been carried out for both reflectance and luminescence measurements.

Our evaluation of the system consists of four parts. Firstly, the sensors and calculation methods are introduced. Secondly, the system design and realization is described. Thirdly, the performance of the system is defined. Lastly, the success of the project is discussed, and ideas are offered for future improvement.

## 2. Experimental Section

Building up the spectral imaging system for a wide range requires a lot of different components. In this section, we introduce the main components of our system and mathematical methods for reflectance and fluorescence calculations.

### 2.1. Cameras

To our knowledge, there is no spectral imaging device available for the 200–2500 nm spectral range. Furthermore, such a wide range cannot be acquired with a single sensor, such as a silicon-based charge–coupled device (CCD)/complementary metal-oxide-semiconductor (CMOS) or indium gallium arsenide (InGaAs) based infrared sensor. However, it is possible to combine different cameras (as listed in Table 2) and achieve the spectral range required. Sensor fusion is a reasonable implementation, as the wavelength ranges overlap nicely. In addition, as the camera sensors measure simultaneously, acting as a single combined camera, the measurement time is effectively one third, when compared to three separate cameras. All cameras used in our development were manufactured by Spectral Imaging Limited (Finland).

### 2.2. Light Sources

In spectral imaging, it is important to have an appropriate light source to illuminate an object. Good properties are smoothness and a continuous spectral power distribution (SPD) over the wavelength range. A halogen lamp is a black body radiator and meets these requirements for the 400–2500 nm wavelength range. The UV region of 200–400 nm has however been challenging, because there has of yet been no light source that has fully, smoothly and continuously covered the whole wavelength range. Usually, measurements have been only performed for several wavelengths, where the light source used has had a satisfying SPD. However, NKT Photonics A/S has recently manufactured a UV supercontinuum light source that meets the previously mentioned requirements at the 270–400 nm wavelength range [9]. In this study, we used an ultraviolet B (UV-B) light source (8 W fluorescent tube F8T5), which has an appropriate SPD in the 309–331 nm wavelength range. This UV light allows us to measure reflectance inside the wavelength range and also gives an accurate spectral range of excitation to measure luminescence.

### 2.3. Reflectance and Luminescence Calculations

Reflectance *R*(*λ*) is calculated from the measured signal *S*(*λ*):

(1) $$R(\lambda )=\frac{S(\lambda )-B(\lambda )}{W(\lambda )-B(\lambda )}$$

where *B*(*λ*) is dark current obtained with a covered objective and *W*(*λ*) is the spectrum of the reference white.

Emission spectrum *E*(*λ*) can be obtained with an excitation light source that does not have any spectral power *W*(*λ*) in the detection range:

(2) $$E(\lambda )=\frac{S(\lambda )-B(\lambda )}{C(\lambda )}$$

where *C*(*λ*) is the wavelength-dependent response of the camera over the wavelength range (we discuss this further on in the article). However, if a light source has some spectral power in the detection range, the emission spectrum can be approximated using:

(3) $$E(\lambda )=\frac{S(\lambda )-W(\lambda )}{C(\lambda )}$$

The latter method for emitted spectrum extraction is not as accurate as the first, because emitted and reflected signals are mixed.

The wavelength-dependent response of the sensor over the wavelength band is not uniform and can be measured either with a calibrated light source or with a reference device, such as a radiometer (the latter requiring a stable light source when measured in conjunction with a sensor). A measured sensor signal from a light source is a product of the light source spectrum, and the wavelength is a dependent response of the sensor. Thus, the final wavelength-dependent response is calculated by dividing the measured spectrum with the corresponding reference spectrum.

The response of the sensor might not be perfectly linear, and this will cause error in the results. Usually, non-linearities occur with digital counts < 10% and >90% of the sensor maximum. This issue can be fixed, for example, with a look-up-table, but it must be undertaken before any other calculations are made.

### 2.4. System Design

**Figure 1.**
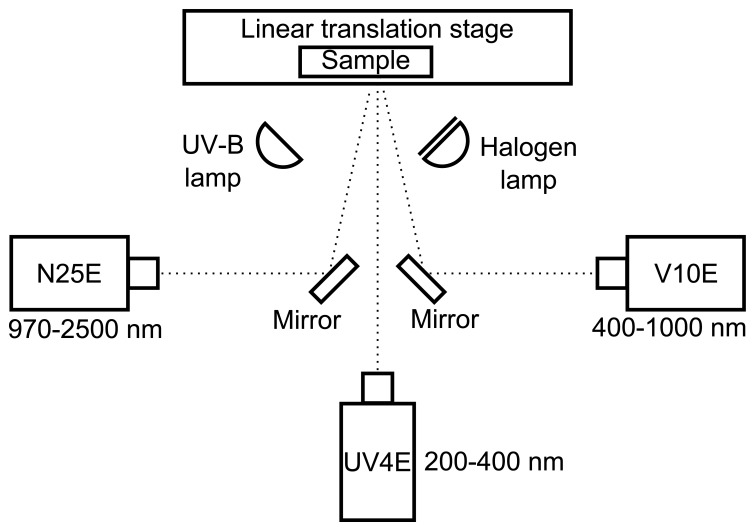
Measuring system: three line spectral cameras are focused on the same line on the sample. The sample is mounted on a linear translation stage for scanning. The UV-B and the halogen lamp provide light for reflectance and luminescence measurements.

Sensor fusion using three line spectral cameras (Table 2) was implemented as presented in Figure 1. In this setup, the ultraviolet camera UV4E is looking directly at the sample. Other cameras are alongside, and silver surface mirrors are used to get sight of the sample. The distances from sample to the camera sensors were approximately 60 cm. The cameras where aligned so that all cameras observed the same spatial line on the sample surface. The alignment was performed accurately, within a few pixels, to ease the pixel matching in the calculations. An alternative option could have been to put the cameras in a row and use dichroic mirrors, which would have made the alignment easier. Unfortunately, we did not manage to find dichroic mirrors with transmissions that would have matched the responses of our cameras (Table 2). For the V10E and UV4E cameras, a spatial binning of three and two pixels, respectively, was used to enhance the signal.

Reflectances of silver surface mirrors were verified with a PerkinElmer Lambda 1050 spectrophotometer to ensure reflectivity over the measuring wavelength range. The Spectralon^®^ white reference was mounted on a linear translation stage to acquire the light source spectrum during measurements. The quality of the Spectralon^®^ was also ensured using a PerkinElmer Lambda 1050 spectrophotometer. In this case, the Spectralon^®^ reference was found to be non-luminescent, even in the UV region, which is a highly important property in reflectance measurements.

The designed system can be extended for imaging bigger samples with the replacement of the linear translation stage with a two-dimensional translation stage, which can move in two dimensions. Therefore, the sample can be moved perpendicularly to the scan direction between scans. This allows us to take a required amount of scans next to the previous one, in order to cover the whole sample. Naturally however, the differently scanned images have to be merged together later.

## 3. Results and Discussion

The developed system consists of many parts, each of which will have an effect on performance. In the following section, we present calibration results for the system, e.g., signal-to-noise ratios and resolutions. We also discuss the few challenges posed by the presented system and their possible solutions.

### 3.1. Peak Signal-to-Noise Ratio

**Figure 2.**
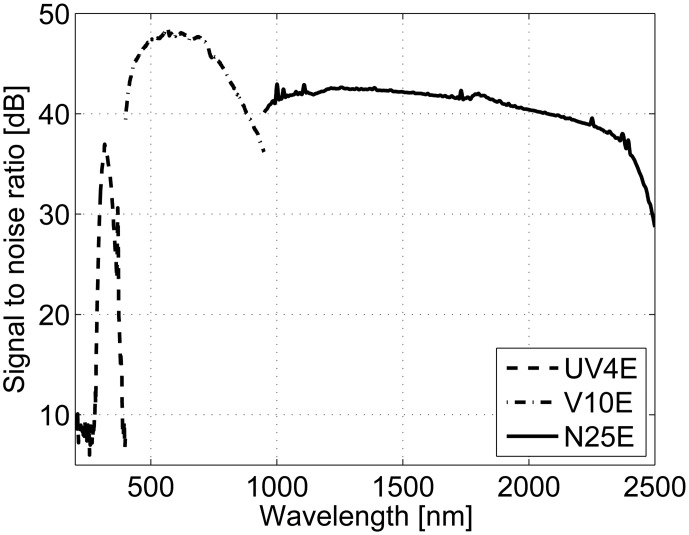
The signal-to-noise ratio (SNR) measurement from the white reference. Two illuminations were used: UV-B for λ < 400 nm and halogen for λ > 400 nm.

The signal-to-noise ratio (SNR) describes the ratio between the desired signal and noise. A higher SNR value indicates a stronger signal compared to the noise. SNR is calculated in decibels:

(4) $$SNR=20\log_{10}(\frac{\mu }{\sigma })$$

where μis the signal mean and σ is the standard deviation of the noise. An SNR value of 10 dB means that there is 10% of the noise in the signal, and 20 dB that amount of noise is 1%. We have measured and calculated SNR values as a function of wavelength for the developed system, and the results are shown in Figure 2. The used exposure times in the measurement were 1000 ms at 200–399 nm, 120 ms at 400–999 nm and 4 ms at 1000–2500 nm. The radiance of the sample was 7.4–126 mW·sr^−1^·m^−^**^2^** at 400–780 nm (Konica Minolta CS–2000 radiometer).

Based on the results in Figure 2, the SNR is over 30 dB in the 297–350 nm, 368–370 nm and 400–2488 nm ranges and over 35 dB in the 309–331 nm and 400–2425 nm ranges.

### 3.2. Spatial Resolution

**Figure 3.**
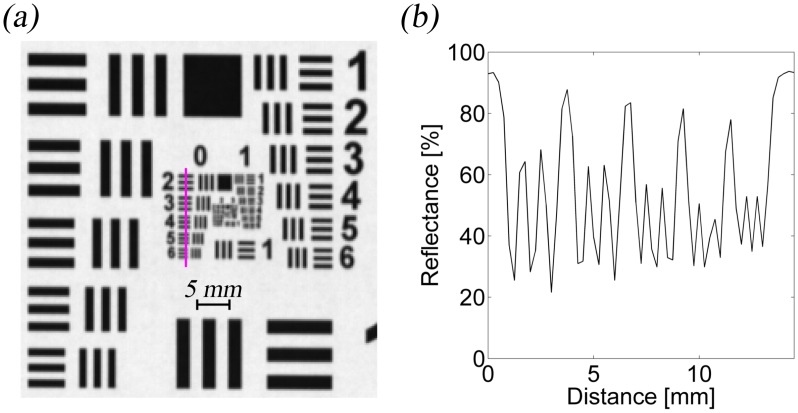
The example of spectral micro-scale imaging capabilities: (**a**) USAF–1951 resolution target at 550 nm; and (**b**) reflectance as a function of distance along the magenta sample line (up to down) from (**a**).

The amount of pixels in the spatial axis of the output image was fixed to be the same (4 px/mm) for all three cameras. The resolution on the object plane was verified with a USAF–1951 resolution target, as shown in Figure 3, and the achieved resolutions are presented in Table 3 in line pairs per millimeter (lp/mm) units. For 4 px/mm, which corresponds to a 250 μm spot size, the maximum resolution is 2 lp/mm. The resolution could be improved with lens system modification.

### 3.3. Spectral Performance

**Figure 4.**
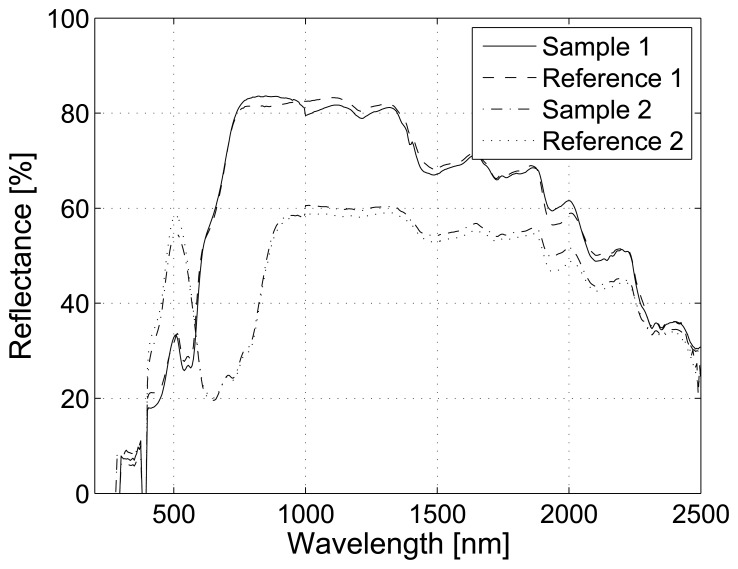
The accuracies of the reflectance measurements. Two samples are measured with the developed system and with the reference device.

**Figure 5.**
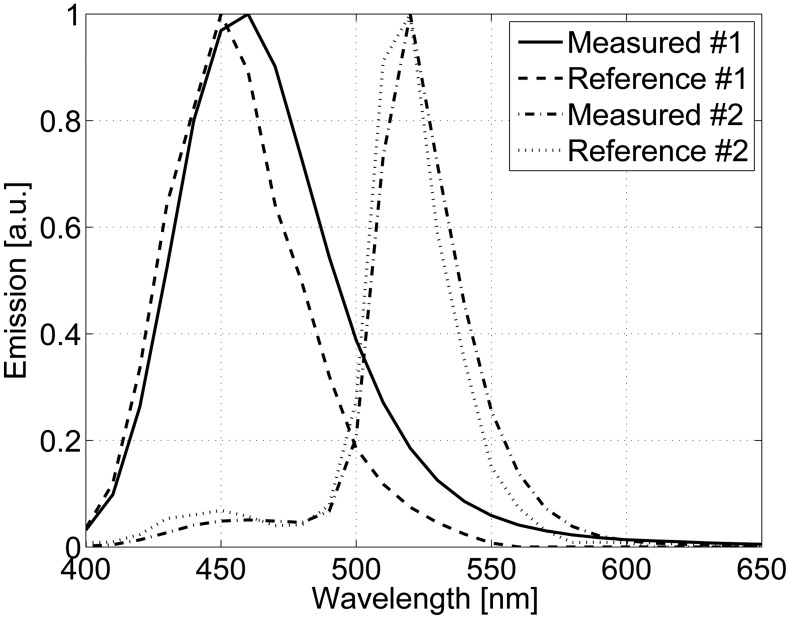
The accuracies of the luminescence measurements. Two samples are measured with the developed system, and the reference is calculated from the reference data for the used light source.

The spectral performance of the developed system was verified with reference measurements. In the case of reflectance, the reference method was a PerkinElmer Lambda 1050 spectrophotometer, and the sample was a mini GretagMacbeth ColorChecker, which had 22 non-fluorescent patches. Luminescence samples were comprised of six Fluorilon™ reflectance materials. These samples were excited with the UV-B light source and the spectrum measured with a Hamamatsu PMA–11 C7473 spectrometer. The excitation spectrum was used to simulate the emission of luminescent samples from reference data. The accuracies of reflectance measurements are presented in Figure 4, where it is visible that the spectra are not perfectly overlapping. The reason for this mismatch is the use of different measuring geometry between methods: the spectrophotometer uses 0°/d geometry, and the developed system is based on 45°/0° or d/0° geometry, when specular reflection affects the results. However, despite the rather large setup difference, the degree of error is very small. Figure 5 shows the accuracies of the luminescent measurements. Here, it can be seen that the results correspond accurately. The highest degree of difference is naturally observed in the ends of the spectra where the measurement devices have lower signal-to-noise ratios (see Figure 2).

**Figure 6.**
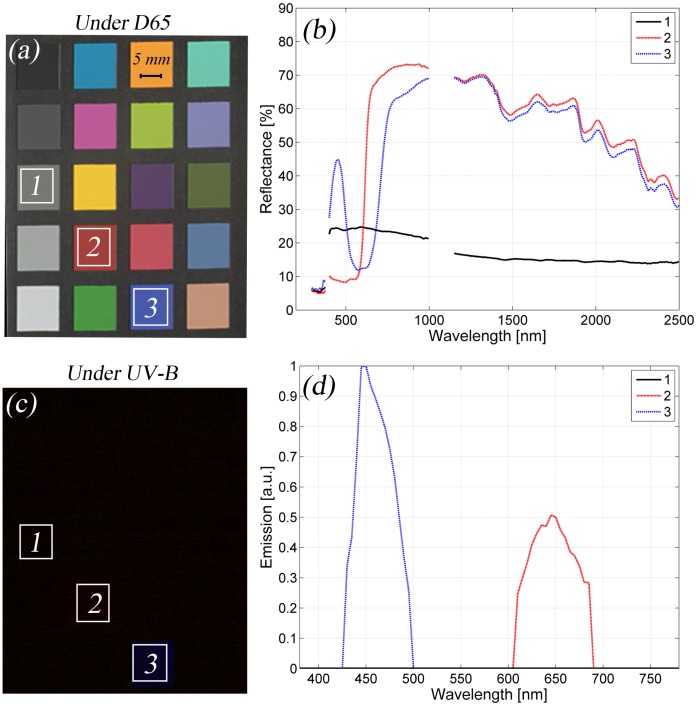
Example results with the developed imaging system: (**a**) the simulated RGB image from the mini ColorChecker under the D65 light source and three sample areas; (**b**) the reflectance spectra of the samples; (**c**) the simulated RGB image from the mini ColorChecker under the UV-B light source; and (**d**) corresponding emission spectra. Spectra were not drawn between 1000–1150 nm, because in this range, the responses of spectral cameras are too low, and the data are not reliable.

Finally, the measurement results for the developed system are presented in Figure 6. The standard red–green–blue (RGB) simulation of the mini ColorChecker under D65 illumination from a measured spectral image is shown in Figure 6a, and three sample areas are marked. In Figure 6b, the reflectance spectra of these samples are presented. The luminescent properties of the mini ColorChecker were discovered with the UV-B light source, and Figure 6c presents the RGB simulation under UV-B illumination. The last two samples are luminescent, as seen from Figure 6d.

With the developed system, it is possible to acquire spectral images from a wide spectral range with reduced time, because three cameras are operating simultaneously as one single camera. Thus, only one scan is enough to acquire all the data, as compared to the situation of three separate cameras, where three scans are required. The degree of time saving is considerable, also when compared to a point measurement device, because one can measure 320 points at once with the current setup. Naturally, this means that only a one dimension scan is required instead of two dimensions. Nevertheless, the system presented in this paper poses some challenges with regard to the measuring time, resolution and signal quality. With the used setup, the exposure times were 1000 ms at 200–399 nm, 120 ms at 400–999 nm and 4 ms at 1000–2500 nm; thus, the ultraviolet range creates a bottleneck in measurements by increasing the measurement duration almost by a factor of ten. This increase in measurement time could be solved with a more sensitive sensor or by increasing the amount of light, although the latter might speed up sample contamination.

The wavelength range is not perfectly covered from 200–2500 nm, and most of the challenges lie in the UV range. Missing light from 330–400 nm could be added by using a UV-A light source, which will cover this wavelength range, but it might make emission extraction harder by mixing the reflectance and emission signals. It is also possible to extend the wavelength range towards shorter wavelengths with a UV-C light source. However, UV-C is more harmful to most of the samples and those people in the same room. One option in the future could be to have a UV supercontinuum light source, as mentioned previously [9]. It is also possible to use several non-overlapping light emitting diodes (LED) to cover the UV range. By LEDs, one could run bispectrometric measurements to extract the excitation-emission matrix, which makes it possible to discriminate reflectance and luminescent information from each other.

The points in the fusion spectrum where sensors change reveal the different measuring geometries of different sensors. It is challenging to completely match the overlapping parts of two spectra. This is mostly due to the different responses of different cameras on the particular spectral areas (see Figure 2) and also the setup geometry. The geometries in the developed system are really close to each other. However, two cameras are positioned on different sides of the sample surface, and the light source is positioned beyond. This problem could be solved by using dichroic mirrors, but it is challenging to find dichroic mirrors with appropriate transmittance and reflection values, when compared to the spectral camera responses we used. To the best of our knowledge, there is no existing imaging system with the same capabilities as that developed by us (see Table 1).

## 4. Conclusions

We have introduced a wide spectral range imaging system, which was tested for reflectance and luminescence measurements. This setup can be widely customized, and after modification, it can also measure transmittance. The spatial resolution of the presented system is straightforwardly scalable. Thus, even big samples can be measured with high resolution, and only a two-dimensional translation stage is required. Higher spatial resolutions could be achieved, even to spot sizes of tens of micrometers with appropriate lens systems. Nonetheless, in the extreme small spot sizes, both the polychromatic lens aberrations and the amount of the produced data could be challenging. However, the ability to image a wide spectral range with reflectance and luminescence is a great advantage in practical applications.

Sensor fusion is a challenging task, and there are a lot of things to consider while setting up a system, e.g., sensor responses and appropriate light sources. Particularly challenging is the performance of imaging in the UV region of the electromagnetic spectrum, because sensor sensitivities and light source outputs are weak. Luminescence and reflectance signal separation can also be challenging; however, UV imaging has great potential in both the fields of reflectance and luminescence, which can both reveal a great deal of information from samples. Thus, in the future, more effort could be directed towards the challenges posed by the UV imaging range of the system presented.

## Figures and Tables

**Table 1.** Broad spectral band imaging systems.

**Wavelength region [nm]**	$\text{Resolution}:\frac{\text{spatial}[mm]}{\text{spectral}[nm]}$	**Spatial pixels**	**Author & year**
400–2400	20,000 *^a^*/10	614	Vane *et al.* 1993 [6]
400–1700	0.16–0.27/2.8–10	1,600,320	Antikainen *et al.* 2007 [7]
350–950	0.5/2–3	290	Heia *et al.* 2007 [5]
365–1100	0.06/10–16	2,048	Klein *et al.* 2008 [8]
405–3000	70,000 *^b^*/10	600	Pieters *et al.* 2009 [3]
380–2500	1,000 *^c^*/3–10	384	Spectral Imaging Ltd. 2012 [4]
200–2500	0.25/2–10	320	In this paper

a At 20 km altitude;

b At 100 km altitude;

c At 660 m altitude.

**Table 2.** Specifications of line spectral cameras.

**Camera**	**UV4E**	**V10E**	**N25E**
Wavelength range [nm]	200–400	400–1000	970–2500
Exposure time [ms]	0.5–1000	0.01–80	0.1–20
Array size [px]	1000 × 1000	1600 × 1200	320 × 256
Spectral resolution [nm]	2	2.8	10
Slit width [μm]	30	30	30
Dynamics [b]	12	12	14
Numerical aperture	F *^a^* /2.8	F/2.4	F/2.0

a Focal length.

**Table 3.** Resolutions on the target sample. lp, line pair.

**Wavelength range** [**nm**]	**Horizontal** [**lp/mm**]	**Vertical** [**lp/mm**]
200–399	1	1.78
400–999	1	1.41
1000–2500	1.78	1.26
